# Effects of dexmedetomidine-ropivacaine assisted combined spinal-epidural anesthesia on neutrophil-lymphocyte ratio and postoperative delirium in elderly patients with intertrochanteric femoral fracture

**DOI:** 10.3389/fphar.2024.1454452

**Published:** 2025-01-30

**Authors:** Lili Bai, Lina Zhao, Fang Jia, Ying Liu, Ping Li

**Affiliations:** Department of Anesthesia, Tianjin Hospital, Tianjin, China

**Keywords:** intertrochanteric femoral fracture, combined spinal epidural anesthesia, dexmedetomidine, ropivacaine, neutrophil/lymphocyte ratio, delirium, cognitive dysfunction, hemodynamics

## Abstract

**Objective:**

Intertrochanteric femoral fracture (IFF) is a public issue in the old. Combined spinal-epidural anesthesia (CSEA) is commonly utilized for lower limb orthopedic surgery in elderly patients. Therefore, this study explored the application of dexmedetomidine (Dex) and ropivacaine (Rop) assisted CSEA in elderly IFF patients.

**Methods:**

Totally 187 elderly IFF patients were assigned into the Rop assisted CSEA (Rop-CSEA), low-dose Dex-Rop assisted CSEA (low Dex and Rop-CSEA) and high-dose Dex-Rop assisted CSEA (high Dex and Rop-CSEA) groups. We compared block effects, hemodynamic indicators [heart rate (HR)/respiratory rate (RR)/mean arterial pressure (MAP)] at time before anesthesia (T0)/skin incision (T1)/10 min postoperatively (T2)/suture postoperatively (T3)/anesthesia recovery (T4), postoperative pain mediator release [substance P (SP)/prostaglandin E2 (PGE2)/5-hydroxytryptamine (5-HT)], neutrophil-lymphocyte ratio (NLR), adverse reactions, delirium and cognitive dysfunction incidence.

**Results:**

Compared with the Rop-CSEA group, low/high Dex and Rop-CSEA groups had shortened onset times, prolonged recovery times in sensory/motor block, elevated HR/RR/MAP, repressed pain mediator release, and reduced postoperative delirium and cognitive dysfunction incidences. HR/RR/MAP exhibited reductions followed by elevations at T2-T4, and SP/PGE2/5-HT levels revealed elevations in all groups postoperatively. NLR level displayed enhancement followed by reduction, and NLR in the low/high Dex and Rop-CSEA groups was abated on postoperative days 1–5. Total incidence of adverse reactions in the high Dex and Rop-CSEA group was enhanced.

**Conclusion:**

Dex and Rop assisted CSEA shortens the onset time of anesthesia, maintains perioperative hemodynamic stability, inhibits pain mediator release, reduces postoperative NLR level and the incidence of delirium and cognitive dysfunction in IFF patients.

## Introduction

The incidence of fractures in the elderly is on the rise and has emerged as a prominent health concern in numerous nations (Court-Brown and McQueen, 2016). Hip fractures in older individuals remarkably contributes to morbidity and mortality, exerting a substantial impact on society (Handoll et al., 2021). Among these, intertrochanteric femoral fracture (IFF) is prevalent among the elderly population and are linked with considerable morbidity, mortality, and a decline in life quality (Yoon et al., 2020). Surgery is acknowledged as a crucial tool in the management of IFF (Cheng and Sheng, 2020). Elderly patients commonly experience degenerative body functions, frequently accompanied by cardiovascular diseases, low tolerance to surgery, abnormal vital signs, and hemodynamic fluctuations during surgery. Besides, they are also susceptible to postoperative complications such as delirium, which places additional demands on nursing staff, extends hospital stays, raises hospitalization expenses, and elevates in-hospital mortality (Albanese et al., 2022; Basques et al., 2015; Neuman et al., 2014). The most frequently employed anesthesia protocol for hip surgery is combined spinal-epidural anesthesia (CSEA), which integrates the advantages of both lumbar and epidural anesthesia, providing effective analgesia, minimal respiratory and circulatory depression, and reduced complications (Chu et al., 2015; Johnson et al., 2016; McIsaac et al., 2018; Perlas et al., 2016). Previous studies have documented that the incorporation of sedative medications into standard anesthesia protocols can suppress sympathetic excitation and decrease catecholamine production, thereby promoting vital sign preservation, enhancing hemodynamic stability, and mitigating postoperative delirium (Shi et al., 2019; Shin et al., 2023; Su et al., 2016; Subramaniam et al., 2019; Yousef et al., 2015). Consequently, the choice of suitable anesthetic medications for aiding CSEA in IFF patients is a crucial consideration for mitigating postoperative pain and minimizing the incidence of complications such as delirium.

Postoperative delirium, which primarily impacts the elderly population, frequently leads to unfavorable patient outcomes, including prolonged hospital stays and clinically impaired functional recovery (Hshieh et al., 2017). The pathophysiology underlying postoperative delirium remains incompletely elicited and may encompass various physiological mechanisms of dysfunction, such as the impact of oxidative stress and neuroinflammation (Maldonado, 2013). Given the potential association between delirium and the inflammatory pathways, it is essential to probe specific inflammatory markers linked to postoperative delirium (Neerland et al., 2016). The neutrophil-lymphocyte ratio (NLR) serves as a biomarker for the systemic inflammatory response (Langley et al., 2021). Moreover, an elevated neutrophil count and an NLR ≥ 3.5 are identified as independent risk factors for postoperative delirium, and NLR may be a promising indicator for predicting delirium in elderly patients undergoing total hip arthroplasty for hip fracture (He et al., 2020). Hence, the quest for appropriate sedative drugs to help CSEA, with the aim of suppressing postoperative neutrophil function and reducing postoperative NLR, may yield advantageous therapeutic outcomes for postoperative delirium in IFF patients.

Ropivacaine (Rop) is an amide local anesthetic that is synthesized in its pure levorotatory form (Beilin and Halpern, 2010). Rop demonstrates anti-inflammatory properties by suppressing the expression and activity of the adhesion molecule CD11b in neutrophils (Zhu et al., 2010). Extensive research has been conducted on the application of Rop in postoperative management of fractures (Cheng et al., 2022; Liu et al., 2022; Oura et al., 2023; Swennen et al., 2017; Wang et al., 2022). In addition, Rop may be bound up with a shorter time of motor function recovery (Malhotra et al., 2016). Nevertheless, further research is warranted to explore the use of Rop in CSEA in IFF patients. On the other hand, dexmedetomidine (Dex) is a highly selective agonist of the alpha2-adrenoceptor, known for its sedative, opioid-sparing, and analgesic effects and suitable for both short- and long-term sedation in the intensive care settings (Keating, 2015). Dex also modulates postoperative NLR (Du et al., 2021). Several studies have assessed the effectiveness and potential negative impacts of Dex as an adjunctive treatment for patients undergoing surgery for femur fractures (Dolma et al., 2018; Gopal and Krishnamurthy, 2018; Nwachukwu et al., 2020). The use of Dex as a local anesthetic adjuvant in femur fracture surgery is linked with a prolonged rescue analgesia (Deng and Yu, 2021). Dex has been shown to effectively decrease the need for narcotic drugs and abate the occurrence of postoperative delirium (Xie and Xie, 2018). Dex mitigates the neurotoxic effects induced by Rop (Xu et al., 2022). Nevertheless, the potential application of Dex and Rop in CSEA for elderly patients with IFF has not yet to be fully elucidated. Based on this context, the study aimed to examine the impacts of Dex and Rop assisted CSEA on postoperative block effect, hemodynamics, pain stress response, NLR, adverse effects, delirium, and cognitive dysfunction incidence in IFF patients, with the objective of identifying a suitable anesthetic drug for assisting CSEA in IFF patients.

## Materials and methods

### Ethics statement

All participants provided their signed informed consent. This study adhered to the principles outlined in the Helsinki Declaration and its amendments, and received approval from the Ethics Committee of Tianjin Hospital (approved number: 2023202). This study was conducted without any disruption to the normal clinical practice.

### Study subjects

**FIGURE 1:**
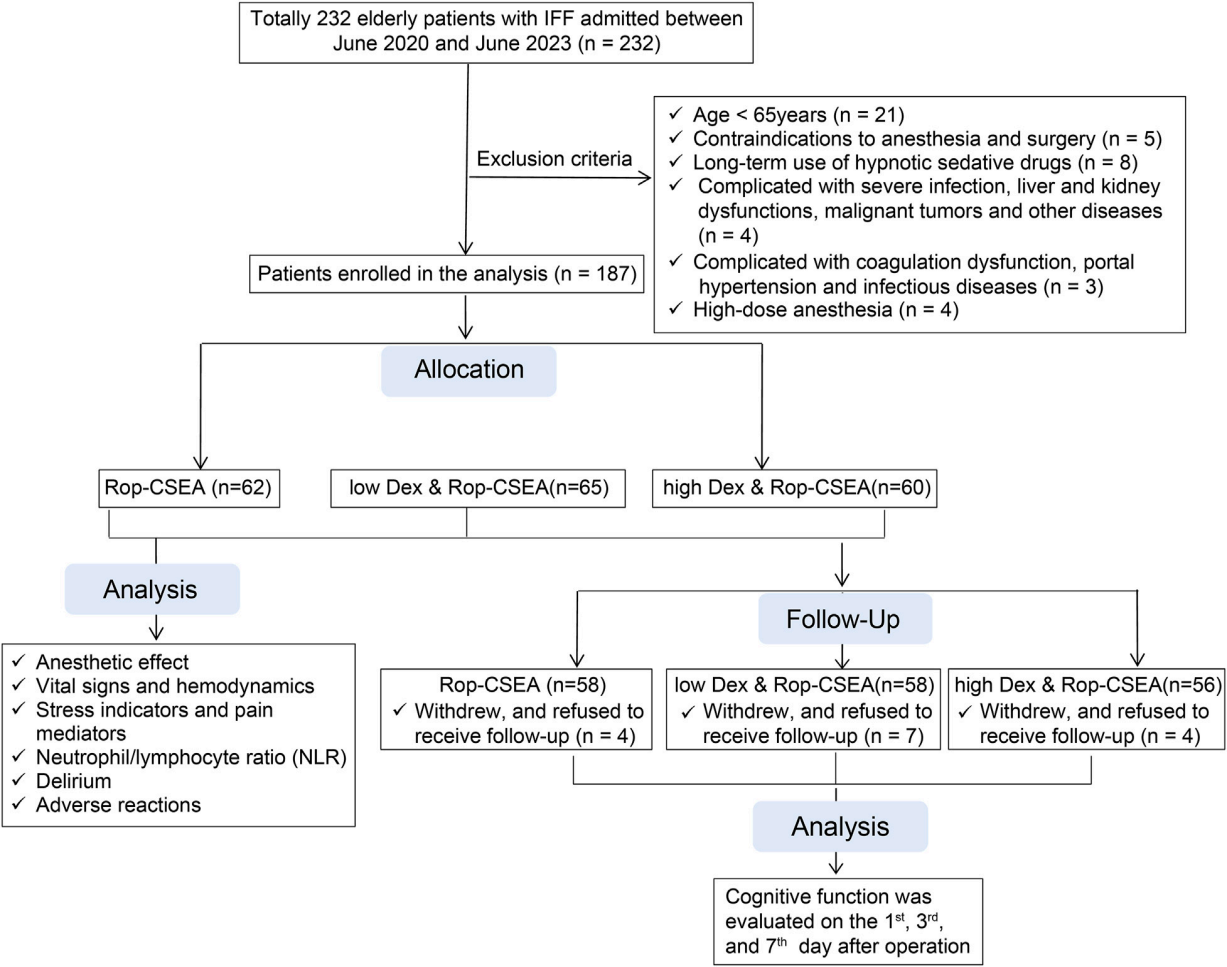
Flow chart for exclusion criteria.

Totally 232 elderly IFF patients admitted to Tianjin Hospital (approved number: 2023202) between June 2020 and June 2023 were included in this study. The inclusion criteria were as follows (Court-Brown and McQueen, 2016): the presence of a well-defined cause of fracture and a confirmed diagnosis of IFF by computed tomography and X-ray examinations (Handoll et al., 2021); age ≥65 years (Yoon et al., 2020); American Society of Anesthesiologists (ASA) class I ∼ II; and (Cheng and Sheng, 2020) the first onset. The exclusion criteria included (Court-Brown and McQueen, 2016): anesthesia and surgical contraindications (Handoll et al., 2021); mental illness, cognitive impairment, hearing impairment, or inability to communicate normally, preoperatively (Yoon et al., 2020); long-term use of hypnotic sedative drugs (Cheng and Sheng, 2020); complications of serious infections, liver and kidney function abnormalities, malignant tumors and other diseases (Albanese et al., 2022); complications of coagulation dysfunction, portal hypertension and infectious diseases (Basques et al., 2015); withdrew and did not accept follow-up (Neuman et al., 2014); high-dose anesthesia (Chu et al., 2015); allergic reaction to the study drugs. In the light of the inclusion and exclusion criteria, 187 elderly patients with IFF were finally enrolled in the study (Figure 1 for the flow chart), and arranged into 3 groups (each received different anesthesia methods) using the random number table method, including the Rop assisted CSEA group (Rop-CSEA, n = 62; Rop-CSEA regimen was performed), the low-dose Dex-Rop assisted CSEA group (low Dex and Rop-CSEA, n = 65; low-dose Dex and Rop-CSEA regimen was implemented), and the high-dose Dex-Rop assisted CSEA group (high Dex and Rop-CSEA, n = 60; high-dose Dex and Rop-CSEA regimen was employed).

### Anesthesia method

All patients underwent routine preoperative anesthesia risk detection and routine fasting prior to surgery.

Rop-CSEA group (n = 62): following the patient’s admission to the operating room, the venous access was established, and oxygen was administered via nasal catheter at a flow of 3 L/min. The blood oxygen saturation, electrocardiogram, and invasive arterial blood pressure were monitored. Next, the patients were in the lateral position, and routinely sterilized. Then, the towel was spread out. Under sterile conditions, a puncture was performed at the L3-4 intervertebral space using an 18 G epidural puncture needle. After penetrating to the epidural cavity, the arachnoid was punctured using a 25 G lumbar puncture needle via the aperture of the epidural puncture needle. The puncture was successful if the cerebrospinal fluid outflowed from the suction catheter was bloodless, airless, and clear. After the withdrawal of the needle core, the arachnoid was administered 3 mL of 0.375% Rop (H20060137, Hengrui Pharmaceuticals, Lianyungang, Jiangsu, China) at a uniform speed of 0.2 mL/s. The lumbar puncture needle was then retracted, and an epidural catheter was inserted through the epidural puncture needle, with a 3 cm catheter remaining in position towards the tip, followed by the removal of the epidural puncture needle. Subsequently, the puncture site was covered with sterilized gauze.

Low Dex and Rop-CSEA gruop (n = 65): after subarachnoid anesthesia, the patients were subjected to an intravenous injection of Dex (H20143195, Guorui Pharmaceutical, Leshan, Sichuan, China) at a rate of 0.5 μg/(kg⋅min) for 10 min, followed by a continuous infusion at 0.25 μg/(kg⋅h) until 10 min prior to the end of surgery (Xiong Jingwei, Li Liping, Zhang Lidong, Liu Yang. Effects of dexmedetomidine-assisted combined spinal and epidural anesthesia on hemodynamics and serum T lymphocyte subsets level in elderly patients with intertrochanteric fracture of femur [J]. Frontiers of Medicine (Electronic version), 2021, 13 (07): 104–108).

High Dex and Rop-CSEA group (n = 60): after subarachnoid anesthesia, the patients were subjected to intravenous infusion of Dex at a rate of 0.5 μg/(kg⋅min) for 10 min, followed by a continuous infusion at 0.40 μg/(kg⋅h). The administration was ceased 10 min before the end of the surgery (Xiong Jingwei, Li Liping, Zhang Lidong, Liu Yang. Effects of dexmedetomidine-assisted combined spinal and epidural anesthesia on hemodynamics and serum T lymphocyte subsets level in elderly patients with intertrochanteric fracture of femur [J]. Frontiers of Medicine (Electronic version), 2021, 13 (07): 104–108).

The adjustment of the anesthesia plane for the three groups was controlled at the T8-T10 level, and the surgery was carried out once the desired plane for the surgery was achieved. During the procedure, various measures such as mask oxygen inhalation, electrocardiogram monitoring, and fluid infusion were implemented to maintain the vital signs. The patients received regular administration of midazolam (H20031037, Nhwa, Xuzhou, Jiangsu, China) for sedation, ephedrine (X20010406, Arker, Chifeng, Inner Mongolia, China) to maintain central arterial pressure ≥30% of the baseline value and systolic blood pressure ≥90 mmHg, and atropine (H34023679, Guorui Pharmaceutical) to maintain heart rate >55 beats/min.

### Indicator observation

(1) Clinical baseline data such as age, sex, body mass index (BMI), comorbidities (hypertension, diabetes mellitus, and coronary artery disease), ASA classification, fracture Evans type [modified Evans (Kyle) type: Types 1–2 Stable and Types 3–4 Unstable (Bedrettin et al., 2022)], fracture cause (traffic accident injury, high fall injury, and fall injury) were acquired at enrollment of patients, as well as the general intraoperative conditions, including anesthesia time, surgery time, and intraoperative blood loss.
(2) Block effect: the onset/recovery time of sensory block, the onset/recovery time of motor block were observed in the three groups.
(3) Hemodynamic indicators such as heart rate (HR), respiratory rate (RR) and mean arterial pressure (MAP) of the three groups at time of before anesthesia (T0), skin incision (T1), 10 min during the surgery (T2), suturing after surgery (T3) and anesthesia recovery (T4) were monitored and recorded using a PHILIPS patient monitor MX550 (Philips Medical Systems, Veenpluis, Netherlands).
(4) Pain stress response indicators: venous blood (3 mL) was acquired from the three groups of patients before (before anesthesia) and after (10 min after surgery) surgery and centrifuged routinely, and the serum and plasma were separated. Levels of plasma prostaglandin E2 (PGE2), 5-hydroxytryptamine (5-HT) and serum substance P (SP) were measured utilizing a BioTek ELx808 absorption light microplate reader combined with enzyme-linked immunosorbent assay (ELISA). The kits were purchased from Sangon (Shanghai, China).
(5) NLR: venous blood (1.5 mL) was obtained from patients in the three groups on the 1^st^ day before surgery and the 1^st^, 3^rd^, and 5^th^ days after surgery. A whole blood cell count (NLR = neutrophil count/lymphocyte count) was performed using a Sysmex XE-5000 automatic blood cell analyzer (Sysmex, Kobe, Japan).
(6) The adverse reactions such as nausea/vomiting, bradycardia, transient hypotension, transient hypertension, and respiratory depression during the perioperative period were recorded in the three groups.
(7) Within 24 h postoperatively, patients were assessed for the occurrence of delirium using the Confusion Assessment Method Chinese Reversion, which consisted of 11 items, with 1-4 points for each item, and a total score of 11–44 points, with a score of >22 being the presence of delirium.
(8) The mental state and cognitive function of patients were evaluated utilizing Mini-Mental State Examination (MMSE) on the 1^st^ day preoperatively and the 1^st^, 3^rd^, and 5^th^ days postoperatively, and the total score of the scale was 0–30 points, with the higher score suggesting better mental state and cognitive function. The incidence of cognitive dysfunction was also calculated within 3 days postoperatively (MMSE score decreased by more than 3 points compared with that before surgery).

### Statistical analysis

Data analysis and graphing in this study were conducted using SPSS 21.0 (IBM, Armonk, NY, United States) and GraphPad Prism 8 (GraphPad Software, San Diego, CA, United States). The normality of distribution in continuous variables was assessed by Shapiro-Wilk (W test). Data were categorized into counting data and measurement data, with counting data expressed as number of cases and percentages. The measurement data were tested for normal distribution using the W test, with measurement data that conformed to normal distribution presented as mean ± standard deviation (SD), and those that did not conform to normal distribution expressed as median [interquartile range]. Comparisons between two groups: the Chi-square test was employed for counting data, the independent sample *t*-test was utilized for measurement data that followed a normal distribution, and the Wilcoxon test was applied for measurement data that did not adhere to normal distribution. The diagnostic efficacy was assessed by measuring the sensitivity, specificity, and area under the receiver operator characteristic (ROC) curve (AUC). The analysis of the difference in AUC was conducted using Medcalc^®^ version 15.0 software (Medcalc Software, Ostend, Belgium). Correlations between diagnostic findings and clinical characteristics were assessed using Chi-square test and linear regression analysis. The unpaired *t*-test or χ^2^ test was used to compare two groups of data. Multi-group data comparisons were conducted using one-way analysis of variance (ANOVA), followed by *post hoc* analyses using Tukey’s test. *p* < 0.05 was accepted as indicative of significant differences.

## Results

### Comparative analyses of the clinical data of subjects

As shown in Table 1, no significant differences in the clinical baseline characteristics, including age, sex BMI, comorbidities (hypertension, diabetes mellitus, coronary artery disease), ASA classification, fracture Evans type, and fracture cause were observed among the three groups (all *p* > 0.05), and there were no notable differences in the intraoperative general conditions, such as operative time and intraoperative blood loss (all *p* > 0.05), which were comparable among the three groups.

**TABLE 1:** Comparative analyses of clinical baseline characteristics of three groups of subjects.

Indicators	Rop-CSEA group (n = 62)	Low dex and Rop-CSEA group (n = 65)	High dex and Rop-CSEA group (n = 60)	*P*_a_	*P*_b_	*P*_c_
Age (years)	69.5 [65.0,74.9]	70.3 [65.0,74.1]	69.9 [65.0,75.6]	0.619	0.384	0.911
Sex (n)						
Male	26	34	29	0.287	0.585	0.722
Female	36	31	31			
BMI (n)						
<24 kg/m^2^	48	41	42	0.085	0.413	0.453
≥24 kg/m^2^	14	24	18			
Hypertension (n)						
Yes	15	13	13	0.670	0.831	0.829
No	47	52	47			
Diabetes mellitus (n)						
Yes	13	20	17	0.230	0.403	0.845
No	49	45	43			
Coronary artery disease (n)						
Yes	18	11	15	0.139	0.686	0.280
No	44	54	45			
ASA classification (n)						
I	30	29	25	0.724	0.473	0.857
II	32	36	35			
Fracture Evans type (n)						
Types 1–2 Stable	40	32	28	0.107	0.068	0.858
Types 3–4 Unstable	22	33	32			
Fracture cause (n)						
Traffic accident injury	6	8	8	0.104	0.114	0.984
High fall injury	10	20	18			
Fall injury	46	37	34			
Surgery time (min)	96.62	97.85	98.08	0.825	0.882	0.534
	[56.46,125.56]	[63.57,129.66]	[59.27,123.36]			
Intraoperative blood loss (mL)	129.23	123.86	129.09	0.958	0.895	0.745
	[80.72,173.22]	[92.26,178.68]	[84.03,173.09]			

Note: Rop-CSEA, ropivacaine-combined spinal-epidural anesthesia; low Dex and Rop-CSEA, low-dose dexmedetomidine-ropivacaine assisted combined spinal-epidural anesthesia; high Dex and Rop-CSEA, high-dose dexmedetomidine-ropivacaine assisted combined spinal-epidural anesthesia; BMI, body mass index; ASA, American Society of Anesthesiologists. The measurement data were tested for normal distribution using the W test method, and those that did not conform to the normal distribution were expressed as median [interquartile range]. Comparisons of counting data between the two groups were performed using the Chi-square test, and comparisons of measurement data that did not fit a normal distribution were conducted using the Wilcoxon test. *P*a indicated Rop-CSEA group compared to low Dex and Rop-CSEA group; *P*b denoted Rop-CSEA group compared to high Dex and Rop-CSEA group; and *P*c indicated low Dex and Rop-CSEA group compared to high Dex and Rop-CSEA group.

### Comparison of block effect among the three groups of patients

The low Dex and Rop-CSEA group and the high Dex and Rop-CSEA group exhibited shorter onset times for both sensory and motor block and longer recovery times for both sensory and motor blocks than the Rop-CSEA group (all *p* < 0.05). There was no statistically significant difference noticed between the low Dex and Rop-CSEA group and the high Dex and Rop-CSEA group (all *p* > 0.05) (Table 2). Overall, Dex and Rop assisted CSEA shortened the onset time of anesthesia and prolonged the time of regional anesthesia.

**TABLE 2:** Comparison of block effect in the three groups (min, Mean ± SD).

Indicators		Rop-CSEA group (n = 62)	Low dex and Rop-CSEA group (n = 65)	High dex and Rop-CSEA group (n = 60)	*P*_a_	*P*_b_	*P*_c_
Sensory block	Onset time	15.04 ± 4.22	13.23 ± 4.05	12.45 ± 4.11	0.038	0.002	0.543
	Recovery time	166.89 ± 20.63	176.15 ± 21.95	180.21 ± 21.60	0.042	0.002	0.541
Motor block	Onset time	12.86 ± 2.76	11.69 ± 1.22	11.02 ± 1.07	0.001	0.000	0.109
	Recovery time	96.58 ± 15.97	105.32 ± 17.36	108.69 ± 16.78	0.010	0.000	0.499

Note: Rop-CSEA, ropivacaine-combined spinal-epidural anesthesia; low Dex and Rop-CSEA, low-dose dexmedetomidine-ropivacaine assisted combined spinal-epidural anesthesia; high Dex and Rop-CSEA, high-dose dexmedetomidine-ropivacaine assisted combined spinal-epidural anesthesia. The measurement data were tested for normal distribution by W test, and the measurement data conforming to the normal distribution were expressed as mean ± SD. One-way ANOVA was used to compare the data among multiple groups, and Tukey’s multiple comparison test was utilized for *post hoc* analysis. *P*a indicated Rop-CSEA group compared to low Dex and Rop-CSEA group; *P*b denoted Rop-CSEA group compared to high Dex and Rop-CSEA group; and *P*c indicated low Dex and Rop-CSEA group compared to high Dex and Rop-CSEA group.

### Comparisons of perioperative hemodynamic indicators in three group of patients

**FIGURE 2:**
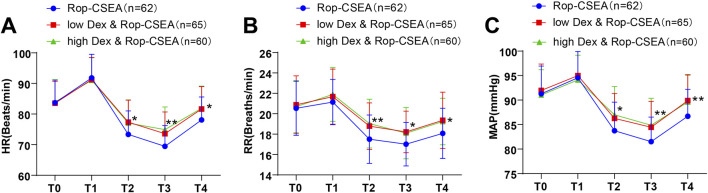
Comparisons of perioperative hemodynamic indicators among three groups of patients. Hemodynamic parameters **(A)** HR **(B)** RR and **(C)** MAP of the three groups at T0, T1, T2, T3 and T4 were compared. Data were presented as mean ± SD. Data comparisons among multiple groups were analyzed by one-way ANOVA, followed by Tukey’s test.*p < 0.05 for comparisons with the Rop-CSEA group at the same time point, and **p < 0.01 for comparisons with the Rop-CSEA group at the same time point.

As presented in Table 3 and Figure 2, at T0, there were no substantial differences in HR, RR and MAP among the three groups (all *p* > 0.05). HR, RR and MAP of the three groups at T1 displayed elevations relative to at T0, with no significant differences in these indicators among the three groups (all *p* > 0.05). At T2-T4, HR, RR and MAP of the three groups revealed reductions followed by increments, and HR, RR and MAP of the low Dex and Rop-CSEA and high Dex and Rop-CSEA groups were raised compared to the Rop-CSEA group (all *p* < 0.05), but with no significant differences noticed between the low Dex and Rop-CSEA and high Dex and Rop- CSEA groups (all *p* > 0.05). The aforementioned results revealed that Dex and Rop assisted CSEA was effective in stabilizing perioperative hemodynamic stability in IFF patients.

**TABLE 3:** Comparisons of perioperative hemodynamic indices among three groups of patients.

Indicators		Rop-CSEA group (n = 62)	Low dex and Rop-CSEA group (n = 65)	High dex and Rop-CSEA group (n = 60)	*P*_a_	*P*_b_	*P*_c_
HR (beats/min)	T0	83.67 ± 7.43	83.55 ± 7.10	83.92 ± 7.34	0.995	0.981	0.957
	T1	91.81 ± 7.71	91.25 ± 7.23	91.04 ± 7.39	0.903	0.831	0.986
	T2	73.55 ± 7.67	77.36 ± 7.20	77.04 ± 7.56	0.006	0.015	0.968
	T3	69.45 ± 6.80	73.58 ± 7.11	74.91 ± 7.40	0.005	0.000	0.569
	T4	78.07 ± 7.52	81.64 ± 7.33	81.88 ± 7.24	0.017	0.012	0.982
RR (breaths/min)	T0	20.53 ± 2.65	20.88 ± 2.84	20.69 ± 2.56	0.704	0.932	0.903
	T1	21.15 ± 2.22	21.67 ± 2.70	21.90 ± 2.65	0.462	0.215	0.862
	T2	17.50 ± 2.37	18.79 ± 2.28	19.03 ± 2.39	0.009	0.002	0.85
	T3	17.01 ± 2.13	18.22 ± 2.04	18.09 ± 2.52	0.016	0.042	0.954
	T4	18.67 ± 2.46	19.36 ± 2.77	19.24 ± 2.28	0.009	0.025	0.96
MAP (mmHg)	T0	91.28 ± 5.67	91.96 ± 5.40	91.03 ± 5.17	0.757	0.964	0.599
	T1	94.55 ± 5.36	94.98 ± 5.28	94.12 ± 5.04	0.895	0.899	0.646
	T2	83.73 ± 5.84	86.25 ± 5.10	86.99 ± 5.79	0.023	0.003	0.723
	T3	81.22 ± 5.01	84.45 ± 5.28	84.87 ± 5.50	0.006	0.002	0.901
	T4	85.69 ± 5.51	89.88 ± 5.32	89.60 ± 5.48	0.003	0.008	0.955

Note: Rop-CSEA, ropivacaine-combined spinal-epidural anesthesia; low Dex and Rop-CSEA, low-dose dexmedetomidine-ropivacaine assisted combined spinal-epidural anesthesia; high Dex and Rop-CSEA, high-dose dexmedetomidine-ropivacaine assisted combined spinal-epidural anesthesia; HR, heart rate; RR, respiratory rate; MAP, mean arterial pressure. The measurement data were tested for normal distribution by W test, and the measurement data conforming to the normal distribution were expressed as mean ± SD. One-way ANOVA was used to compare the data among multiple groups, and Tukey’s multiple comparison test was utilized for *post hoc* analysis. *P*a indicated Rop-CSEA group compared to low Dex and Rop-CSEA group; *P*b denoted Rop-CSEA group compared to high Dex and Rop-CSEA group; and *P*c indicated low Dex and Rop-CSEA group compared to high Dex and Rop-CSEA group.

### Comparisons of preoperative and postoperative pain stress indicators among three groups of patients

**FIGURE 3:**
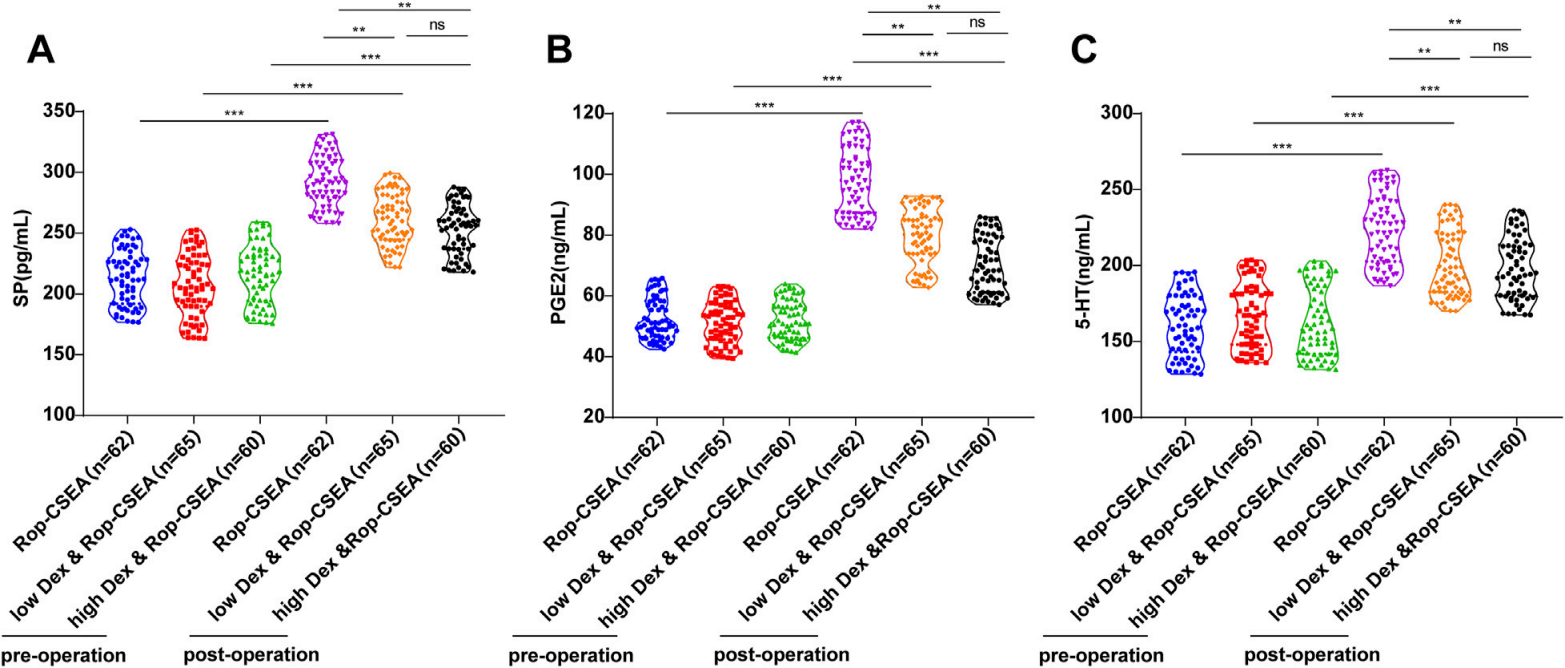
Comparisons of preoperative and postoperative pain stress response indicators in three groups of patients. Comparisons of pain stress response indicators **(A)** SP **(B)** PGE2 and **(C)** 5-HT preoperatively (Preop) and 10 min postoperatively (Postop) in the three groups. Data were expressed as mean ± SD. One-way ANOVA was conducted for data comparisons among multiple groups, and Tukey’s multiple comparison test for *post hoc* analysis. **p < 0.01.

It has been documented that SP, PGE2, and 5-HT are pain mediators that have been linked to the initiation and exacerbation of pain and are frequently used to evaluate the pain stress response in individuals undergoing surgery (Ma et al., 2019). As reflected by ELISA results, there were no prominent differences in SP, PGE2 and 5-HT levels among the three groups before surgery (all *p* > 0.05), and the levels in the three groups were elevated compared to before surgery (all *p* < 0.05). Pain mediator release was suppressed in both the low Dex and Rop-CSEA and high Dex and Rop-CSEA groups versus the Rop-CSEA group, postoperatively (all *p* < 0.01), whereas there were no observable differences between the low Dex and Rop-CSEA and high Dex and Rop-CSEA groups (all *p* > 0.05) (Figure 3). Collectively, Dex and Rop assisted CSEA relieved the stress stimulation of pain on IFF patients.

### Comparison of preoperative and postoperative NLR in three groups of patients

**FIGURE 4:**
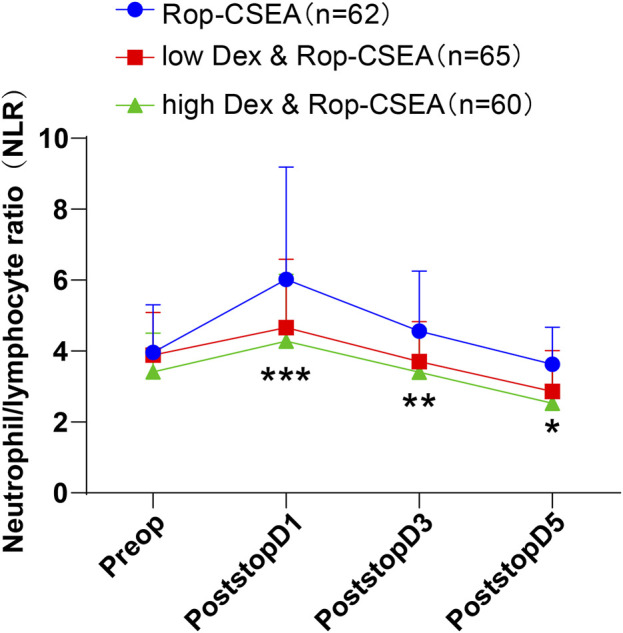
Comparisons of hemodynamic parameters at different times in three groups of patients. Changes in NLR level in the three groups Preop and Postop on days 1 (PostopD1), 3 (PostopD3), and 5 (PostopD5). Values were presented as mean ± SD, with data comparisons among multiple groups analyzed by one-way ANOVA, followed by Tukey’s test. *p < 0.05: compared with the Rop-CSEA group at the same time point, **p < 0.01: compared with the Rop-CSEA group at the same time point, and ***p < 0.001: compared with the Rop-CSEA group at the same time point.

We compared the changes in NLR level in IFF patients in three groups preoperatively and postoperatively on days 1, 3, and 5 (Figure 4), with the results showing that there was no statistically significant difference observed in the preoperative level of NLR among the three groups (all *p* > 0.05). During postoperative days 1–5, NLR level demonstrated a pattern of initial enhancement followed by reduction in the three groups. Notably, the NLR value in the low Dex and Rop-CSEA and high Dex and Rop-CSEA groups was reduced versus the Rop-CSEA group (all *p* < 0.05). There was no notable difference between the low Dex and Rop-CSEA and high Dex and Rop-CSEA groups (all *p* > 0.05). The above results showed that Dex and Rop assisted CSEA was effective on reducing postoperative NLR level in IFF patients.

### Comparison of the incidence of perioperative adverse reactions among the three groups of patients

As listed in Table 4, there was no distinct disparity in the total incidence of adverse reactions in IFF patients in the Rop-CSEA and low Dex and Rop-CSEA groups (all *p* > 0.05), while the total incidence of adverse reactions in IFF patients in the high Dex and Rop-CSEA group was elevated relative to the Rop-CSEA and low Dex and Rop-CSEA groups (all *p* < 0.05).

**TABLE 4:** Comparison of the incidence of perioperative adverse reactions among the three groups (n,%).

Adverse reactions	Rop-CSEA group (n = 62)	Low dex and Rop-CSEA group (n = 65)	High dex and Rop-CSEA group (n = 60)	*P*_a_	*P*_b_	*P*_c_
Nausea/vomiting	1	2	1	—	—	—
Bradycardia	3	1	5	—	—	—
Transient low blood pressure	2	2	7	—	—	—
Transient hypertension	1	1	2	—	—	—
Respiratory depression	1	1	3	—	—	—
Total occurrence	8 (12.90%)	7 (10.77%)	18 (30%)	0.787	0.027	0.013

Note: Rop-CSEA, ropivacaine-combined spinal-epidural anesthesia; low Dex and Rop-CSEA, low-dose dexmedetomidine-ropivacaine assisted combined spinal-epidural anesthesia; high Dex and Rop-CSEA, high-dose dexmedetomidine-ropivacaine assisted combined spinal-epidural anesthesia. Counting data were compared between the two groups using the Chi-square test. *P*a indicated Rop-CSEA group compared to low Dex and Rop-CSEA group; *P*b denoted Rop-CSEA group compared to high Dex and Rop-CSEA group; and *P*c indicated low Dex and Rop-CSEA group compared to high Dex and Rop-CSEA group.

### Comparisons of the incidence of postoperative delirium and cognitive function among three groups of patients

**FIGURE 5:**
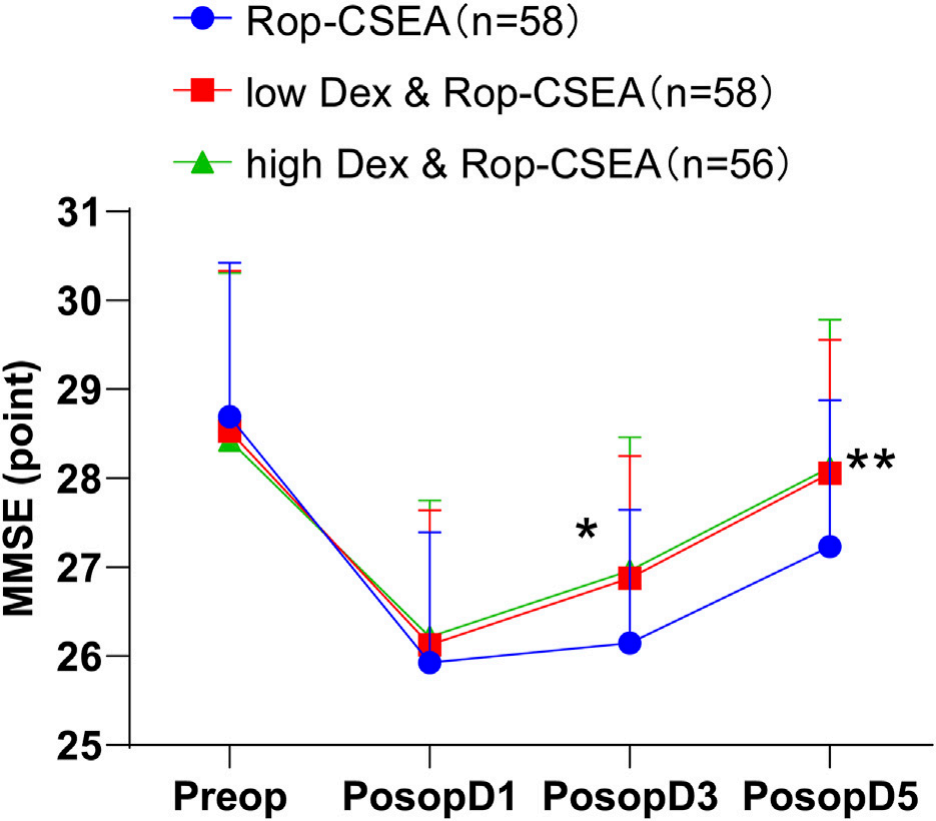
Comparison of postoperative cognitive function in three groups of patients. Changes in MMSE score of patients in the three groups Preop and Postop at PosopD1, PosopD3, and PosopD5. Data were expressed in the form of mean ± SD, and data comparisons among multiple groups were analyzed by one-way ANOVA, followed by Tukey’s test. *p < 0.05: compared with the Rop-CSEA group at the same time point, and **p < 0.05: compared with the Rop-CSEA group at the same time point.

As shown in Table 5 and Figure 5, the incidence of postoperative delirium was decreased in both the low Dex and Rop-CSEA (7.69%) and high Dex and Rop-CSEA groups (13.33%) versus the Rop-CSEA group (20.97%) (all *p* < 0.05), with no substantial difference between the low Dex and Rop-CSEA and high Dex and Rop-CSEA groups (*p* > 0.05). Additionally, there was no significant difference in preoperative and postoperative day 1 MMSE score among the three IFF groups (all *p* > 0.05). On postoperative days 1, 3, and 5, the MMSE score of patients in the three groups presented a reduction followed by an elevation. On the 3^rd^ and 5^th^ days after surgery, the MMSE score of the low Dex and Rop-CSEA and high Dex and Rop-CSEA groups were signally higher than that of the Rop-CSEA group (all *p* < 0.05), but the Low Dex and Rop-CSEA and high Dex and Rop-CSEA groups did not differ significantly (*p* > 0.05). The incidence of cognitive dysfunction within 3 days after surgery was reduced in both the low Dex and Rop-CSEA (7.69%) and high Dex and Rop-CSEA groups (5.00%) compared with the Rop-CSEA group (24.19%) (all *p* < 0.05). Collectively, Dex and Rop-CSEA was effective on reducing the incidence of postoperative delirium and cognitive dysfunction in IFF patients.

**TABLE 5:** Comparisons of the incidence of postoperative delirium and cognitive function among three groups of patients.

Indicators	Rop-CSEA group (n = 62)	Low dex and Rop-CSEA group (n = 65)	High dex and Rop-CSEA group (n = 60)	*P*_a_	*P*_b_	*P*_c_
Delirium; n (%)	13 (20.97%)	5 (7.69%)	2 (13.33%)	0.042	0.005	0.442
MMSE score (Mean ± SD)						
Before surgery	28.69 ± 1.73	28.53 ± 1.80	28.43 ± 1.88	0.839	0.641	0.935
1 day after surgery	25.93 ± 1.46	26.13 ± 1.51	26.22 ± 1.53	0.76	0.575	0.947
3 days after surgery	26.15 ± 1.50	26.88 ± 1.37	26.96 ± 1.50	0.028	0.014	0.958
5 days after surgery	27.03 ± 1.65	28.06 ± 1.50	28.12 ± 1.66	0.01	0.006	0.976
Cognitive dysfunction; n (%)	15 (24.19%)	5 (7.69%)	3 (5.00%)	0.014	0.004	0.719

Note: Rop-CSEA, ropivacaine-combined spinal-epidural anesthesia; low Dex and Rop-CSEA, low-dose dexmedetomidine-ropivacaine assisted combined spinal-epidural anesthesia; high Dex and Rop-CSEA, high-dose dexmedetomidine-ropivacaine assisted combined spinal-epidural anesthesia. The comparisons of counting data between two groups were conducted using Chi-square test. Measurement data that conformed to a normal distribution were presented as mean ± SD. One-way ANOVA analysis was applied for data comparisons among multiple groups, and Tukey’s multiple comparison test for *post hoc* analysis. *P*a indicated Rop-CSEA group compared to low Dex and Rop-CSEA group; *P*b denoted Rop-CSEA group compared to high Dex and Rop-CSEA group; and *P*c indicated low Dex and Rop-CSEA group compared to high Dex and Rop-CSEA group.

## Discussion

Hip fractures have garnered considerable attention on a global scale due to the rapid increase in the geriatric population (Chang et al., 2020). IFF accounts for approximately half of all hip fractures, which are typically led by low-energy mechanisms, such as, a fall from standing height (Ahn and Bernstein, 2010). Surgical intervention is the most effective approach for managing IFF, as it facilitates prompt rehabilitation and functional restoration (Nherera et al., 2018). It is imperative to explore potential strategies for mitigating comorbidities and postoperative mortality of IFF patients (Borges et al., 2014). Our study emphasized that Dex and Rop assisted CSEA shortened the onset time of anesthesia, preserved perioperative hemodynamic stability, suppressed pain mediator release, and lowered postoperative NLR levels and incidence of delirium and cognitive dysfunction in IFF patients.

The intravenous administration of Dex has been linked to an extended sensory block during CSEA (Choi et al., 2020). Rop offers a more distinct block when administered epidurally, enabling a clearer differentiation between sensory and motor block (Chen et al., 2021; Pavlica et al., 2022). Notably, it has been reported that 5 μg Dex and 2.5 mL Rop results in earlier sensory blockade and prolongs both sensory and motor blockade durations in patients undergoing intrathecal anesthesia, without inducing sedation (Ravipati et al., 2017). The utilization of Dex as a supplementary agent in epidural anesthesia for orthopedic femoral fracture surgery has been demonstrated to reduce the onset time of sensory and motor block, extend the duration of analgesia, and prolong anesthesia (Akhondzadeh et al., 2023). The findings of our study indicated that individuals in the low Dex and Rop-CSEA and high Dex and Rop-CSEA groups had reduced onset time for sensory and motor blocks, as well as extended recovery time for sensory and motor blocks, suggesting that Dex and Rop assisted CESA accelerated the onset of anesthesia and prolonged the duration of regional anesthesia.

Dex mitigates diverse stress responses during surgical procedures and sustains hemodynamic stability when employed as a supplement to general anesthesia (Chavan et al., 2016). Dex demonstrates superior clinical efficacy in enhancing perioperative hemodynamics among elderly gynecological patients undergoing laparoscopic surgery (Li Q. et al., 2023). Rop is effective in providing anesthesia for CSEA in caesarean sections, and is recommended due to its minimal impact on hemodynamics, shorter sensory and motor block time, and low occurrence of adverse reactions (Wang et al., 2019). MAP and HR in patients who received Dex and Rop were observably reduced relative to patients who received Rop alone (Lin et al., 2013). Consistently, our study found that HR, RR and MAP were higher in the low Dex and Rop-CSEA and high Dex and Rop-CSEA groups than in the Rop-CSEA group, indicating that Dex and Rop assisted CSEA effectively stabilized perioperative hemodynamic stability in IFF patients.

SP, PGE2, and 5-HT are pain mediators implicated in the onset and exacerbation of pain (Ma et al., 2019). SP is a neuropeptide known for its injury-stimulating capability, which is widely distributed in the systemic system, and can exacerbate pain by facilitating 5-HT release and also contributes to the transmission of the pain signals (Saini et al., 2020). PGE2 is implicated in inflammation, fever, pain, and inflammatory diseases (Koeberle and Werz, 2015). 5-HT plays pivotal roles in the central nervous system, such as regulating pain tolerance and mood stability (Viguier et al., 2013). PGE2 level in the joint fluid of patients treated with Dex and Rop is abated at 6, 12, 24, and 48 h post-surgery (Li et al., 2017). The coadministration of Dex with Rop results in a notably extended period of postoperative analgesia and reduced need for postoperative analgesics (Balasubramaniam et al., 2023). Innovatively, our study results revealed that the low Dex and Rop-CSEA and high Dex and Rop-CSEA groups repressed SP, PGE2, and 5-HT releases, showing that Dex and Rop assisted CSEA relieved the stressful stimulus of pain in IFF patients.

It has been demonstrated that surgical stress produced by the surgery can cause cellular immunosuppression, disrupt the neuro-endocrine-immune network, and activate a great number of inflammation-associated cells in the body. NLR is a widely accessible, easily calculable, and replicable indicator of inflammation (Zhou et al., 2024). Upon activation, neutrophils can facilitate the release of myeloperoxidase, proteolytic enzymes, and oxygen, which in turn cause damage to the blood-brain barrier, inflict damage to brain, accelerate apoptotic rate, thereby inducing delirium (Wu et al., 2023; Zhou et al., 2024). The increment of NLR postoperatively may serve as a predictive indicator for mortality in patients undergoing hip fracture surgery (Wu et al., 2023). Anesthesia can reduce the corresponding immunosuppression by reducing the surgical stress response. Our results suggested that Dex and Rop-CSEA was effective on reducing postoperative NLR level in IFF patients. The possible reason is that Dex can further reduce surgical stress-caused immunosuppression by inhibiting parasympathetic nerve, cell metabolism and immune response, which is conducive to reducing the increase of NLR. The utilization of various anesthesia techniques or adjuvant medications has the potential to alleviate stress response to surgical procedures, mitigate adverse reactions, and enhance overall clinical outcomes (Li Y. et al., 2023). Dex is a dextroisomer and is a highly selective α2 adrenergic receptor agonist. Upon action on the human body, Dex primarily exerts its effects through the medullary vasomotor center and so on, and its burden and effect on the liver and kidney are small. Therefore, it contributes to a low incidence of adverse reactions (Hong Xiaoya, Yao Bin, Li Yangyang. Effects of dexmedetomidine-assisted spinal epidural block anesthesia on stress response and cognitive function in elderly patients with intertrochanteric fracture [J]. Clinical Research and Practice, 2023, 8 (Zhu et al., 2010): 21–24). Additionally, Dex maintains hemodynamic equilibrium, reduces the requirement for anesthetic agents, and has a relatively mild impact on respiration (Hall et al., 2016). However, adverse reactions, specifically bradycardia and hypotension, were reported in patients who received Dex and Rop (Djaiani et al., 2016). Our study did not reveal a significant increase in the overall incidence of adverse reactions in IFF patients in the low Dex + Rop-CSEA group compared to Rop-CSEA, but the high Dex + Rop-CSEA group exhibited an elevated total incidence of adverse reactions, specifically an increase in the incidence of bradycardia and transient hypotension. The results suggested the risk of high-dose Dex in assisting CSEA applications. Caution is advised for patients with preexisting hypovolemia or cardiac conduction block, and co-administration should be considered if necessary.

Elderly patients with thoracolumbar fracture are affected by stress reactions during surgery, and the body will secrete a large amount of inflammatory mediators, which are prone to cause certain damage to the central nervous system to affect the recovery of postoperative cognitive function and induce delirium (Vasunilashorn et al., 2015; Hall et al., 2016). Dex not only decreases the frequency of postoperative delirium in elderly patients following cardiac surgery, but also delays its onset and shortens its duration (Djaiani et al., 2016). In elderly patients, the administration of intravenous Dex postoperatively for 12 h (0.2 μg/kg/h) potentially improves recovery quality and enhances postoperative cognitive function (Guo et al., 2015). Local Rop mitigates surgery-caused impairments in trace and context memory (Koyama et al., 2019). Dex and Rop treatment effectively improves cognitive function, alleviates pain, and reduces the inflammatory factor level (Liu et al., 2021). Intriguingly, our results suggested that Dex and Rop assisted CSEA was effective on reducing the incidence of postoperative delirium and cognitive dysfunction in IFF patients. The possible reason is that Dex can stimulate the α2 receptors on the medulla oblongata and pons and reduce the secretion of norepinephrine and the stress response of the patients’ organism, thereby reducing the release of inflammatory mediators and exerting a cerebroprotective effect that may prevent the onset of postoperative delirium (Tang et al., 2020; Bao and Tang, 2020). Furthermore, Dex has been shown to inhibit the expression levels of inflammatory factors and reduce the inhibitory effects of inflammatory mediators on neurons in the hippocampus, thus protecting patients’ cerebral nerves and lowering the incidence of postoperative delirium (Wang and Wang, 2021; Lv et al., 2020). It has been documented that Dex is involved in neuronal growth, proliferation and differentiation in the cerebral cortex, which contributes to the maintenance of neural function stability in patients’ brains, thus effectively preventing the occurrence of delirium, cognitive dysfunction and other related complications (Endesfelder et al., 2017).

Taken together, our study highlighted that Dex and Rop assisted CSEA shortened the onset time of anesthesia, maintained perioperative hemodynamic stability, inhibited pain mediator release, reduced postoperative NLR level and the incidence of delirium and cognitive dysfunction in IFF patients. Nevertheless, our study had certain limitations. Firstly, the sample size of this study was limited, especially in the detection of differences in adverse effects and rare outcomes, and further expansion of the sample size would enhance the generalizability of the findings. The single-center design of this study may restrict the generalizability of its findings to other hospitals or regions with different patient demographics and clinical practices. Multi-center studies are necessary to validate the generalizability of the results across diverse populations and healthcare environments. Secondly, further observations were needed to track changes in NLR levels at additional time points. Thirdly, Our study focuses on immediate and short-term postoperative outcomes; however, it does not provide data regarding long-term effects, such as chronic pain or persistent cognitive dysfunction. Long-term follow-up would be valuable in evaluating the sustained benefits or potential late-onset complications of Dex-Rop assisted CSEA. Furthermore, there was a need for further exploration of the use of alternative sedative-anesthetic drug combinations to aid in CSEA in IFF patients. Additionally, there were no significant differences in comorbidities or baseline cognitive function among the three groups of participants included in this study. Potential variability in patient characteristics may affect the reproducibility and reliability of the study results, and controlling for these variables or providing a more detailed analysis can strengthen the conclusions. While the study demonstrates beneficial effects of Dex on postoperative outcomes, the underlying mechanisms, particularly how Dex reduces NLR and the incidence of delirium, are not fully elucidated. Further research into the mechanistic pathways can provide deeper understanding and aid in refining anesthesia protocols.

## Data Availability

The raw data supporting the conclusions of this article will be made available by the authors, without undue reservation.
